# Characterization of Progranulin Gene Mutations in Portuguese Patients with Frontotemporal Dementia

**DOI:** 10.3390/ijms25010511

**Published:** 2023-12-29

**Authors:** Maria Rosário Almeida, Miguel Tábuas-Pereira, Inês Baldeiras, Marisa Lima, João Durães, João Massano, Madalena Pinto, Catarina Cruto, Isabel Santana

**Affiliations:** 1 CNC—Center for Neuroscience and Cell Biology, University of Coimbra, 3004-504 Coimbra, Portugal; ines.baldeiras@sapo.pt (I.B.); isabeljsantana@gmail.com (I.S.); 2 Neurology Department, Centro Hospitalar e Universitário de Coimbra, 3004-561 Coimbra, Portugal; miguelatcp@gmail.com (M.T.-P.); marisalima5@hotmail.com (M.L.); duraes.jlo@gmail.com (J.D.); 3 Faculty of Medicine, University of Coimbra, 3000-370 Coimbra, Portugal; 4 Neurology Department, Centro Hospitalar Universitário de São João, 4200-319 Porto, Portugal; jmassano@med.up.pt (J.M.); madalena.pinto@chsj.min-saude.pt (M.P.); 5 Neurology Department, Hospital Pedro Hispano, Unidade Local de Saúde de Matosinhos, 4464-513 Matosinhos, Portugal; caticruto@gmail.com

**Keywords:** frontotemporal dementia, *GRN* mutations, mechanism of haploinsufficiency, low PGRN levels

## Abstract

In Portugal, heterozygous loss-of-function mutations in the progranulin (*GRN*) gene account for approximately half of the genetic mediated forms of frontotemporal dementia (FTD). *GRN* mutations reported thus far cause FTD through a haploinsufficiency disease mechanism. Herein, we aim to unveil the *GRN* mutation spectrum, investigated in 257 FTD patients and 19 family members from the central/north region of Portugal using sequencing methods. Seven different pathogenic variants were identified in 46 subjects including 40 patients (16%) and 6 relatives (32%). bvFTD was the most common clinical presentation among the *GRN* mutation patients, who showed a global pattern of moderate-to-severe frontotemporoparietal deficits in the neuropsychological evaluation. Interestingly, two mutations were novel (p.Thr238Profs*18, p.Leu354Profs*16), and five were previously described, although three of them only in the Portuguese population, suggesting a population-specific *GRN* mutational spectrum. The subjects harboring a *GRN* mutation showed a significant reduction in serum PGRN levels, supporting the pathogenic nature of these variants. This work broadens the mutation spectrum of *GRN* and the identification of the underlying *GRN* mutations provided an accurate genetic counselling and allowed the enrolment of subjects with *GRN* mutations (both asymptomatic and symptomatic) in ongoing clinical trials, which is essential to test new drugs for the disease.

## 1. Introduction

Frontotemporal dementia (FTD) is the second most common presenile type of dementia after Alzheimer’s disease (AD) with an estimated prevalence, in the United States, ranging between 15 and 22 cases per 100,000 individuals aged 45–64 years and an incidence (new cases per 100,000 person-years) ranging from 2.7 to 4.1 [1]. Notably, sixty percent of FTD cases are diagnosed in individuals between the ages of 45 and 64, with an additional 10% presenting at <45 years of age and 30% presenting at >65 years of age [1,2]. Interestingly, in Europe, the estimated number of new FTD cases per year was 12,057, constituting a substantial burden on the European health and welfare system [3]. According to this recent multinational European study, FTD is more common than previously recognized, and diagnosis should be considered at any age [3].

Affected individuals usually present gradual changes in behavior, social awareness, and language. There are three main clinical subtypes of FTD: behavioral variant frontotemporal dementia (bvFTD), in which changes in behavior and social conduct predominate, including loss of social awareness and poor impulse control; semantic dementia (SD), with loss of long-established knowledge of words, objects, anomia resulting in impaired word comprehension [4,5]; and progressive non-fluent aphasia (PNFA) in which patients progressively lose fluency of speech, resulting in agrammatism and non-fluent speech with intact word comprehension [6,7,8]. In a large multicenter study, the frequencies of the three subtypes were estimated at 57% for bvFTD, 24% for PNFA, and 19% for SD [9]. In addition to these classical presentations, FTD patients may also develop symptoms of motor neuron disease (MND) and atypical parkinsonian syndromes such as: corticobasal degeneration (CBD) and progressive supranuclear palsy (PSP) [10,11,12,13].

About half of the patients show a family history of the disease, and one-third of those carry an autosomal dominant mutation in three main genes: microtubule associated protein tau (*MAPT*) [14], progranulin (*GRN*) [15,16], and the chromosome 9 open reading frame 72 (C9orf72) gene [17,18]. In a large international study, it was concluded that the pooled mutation frequencies of these genes are *C9orf72* 42.1%, *GRN* 34.6%, and *MAPT* 23.2%, although geographical variability was observed in the distribution of these mutations across the world and even across European populations [19]. Indeed, individuals with *GRN* mutations were more common than those of other groups in Italy, Spain, and Belgium, whereas individuals with *MAPT* mutations were found more frequently in the Netherlands and in the US west coast. Interestingly, in Portugal, *GRN* mutations are almost as common as *C9orf72* mutations, accounting for half of the genetic mediated forms of FTD [19]. Thus far, more than 100 pathogenic variants in the *GRN* gene are reported in the Human Gene Mutation Database (HGMD) and include frameshift, nonsense and splice-site mutations which result in premature stop codon insertion and nonsense-mediated RNA decay, producing null alleles [15,16,20]. These null allele mutations result in loss-of-functional PGRN, and haploinsufficiency appears to be the mechanism underlying the disease in these patients [21,22]. In fact, a significant reduction in PGRN levels is observed in biological fluids such as the cerebrospinal fluid (CSF), plasma, and serum of patients with *GRN* mutations, even in unaffected mutation carriers [23]. Thus, elevating and/or restoring PGRN levels, through multiple mechanisms of action, may prevent or slow down disease progression [24,25]. Following this rationale, new disease-modifying therapies that aim to normalize PGRN deficiency are now underway in clinical trials for individuals with *GRN* mutations. These approaches include blocking the degradation pathway of PGRN using monoclonal antibody therapy, gene therapy approaches to replace the null-allele using viral vectors, blood–brain barrier transport vehicles for protein replacement therapy, and small-molecule histone deacetylase inhibitors that increase the expression of PGRN [25,26]. Thus, the recent studies in FTD on fluid, neuroimaging, or cognition biomarkers will undoubtedly prove to be crucial in monitoring the treatment response and guiding therapeutic development [27,28]. Therefore, it is extremely important to disclose the *GRN* gene mutations of specific populations, particularly in countries like Portugal where this gene accounts for such a high proportion of the genetic mediated cases of the disease. In addition, identifying the individuals harboring *GRN* mutations at the presymptomatic stage of the disease is crucial for the selection of cases for clinical trials, as the optimal window of opportunity for treatment might be small in *GRN* mutation carriers. Actually, recent longitudinal studies have suggested that the time-window between emerging pathophysiological changes and the first clinical symptoms is short in *GRN* mutation carriers and covers only 2–4 years [29,30]. Thus, because of the fast progression of pathophysiological changes, any disease-modifying treatment should be started very early in the disease process in order to be effective. Hence, in the present study we aim to unveil the *GRN* mutation spectrum of Portuguese FTD patients and at-risk family members. In addition, the clinical and neuropsychological evaluations of *GRN* mutated patients will be presented.

## 2. Results

**Figure 1:**
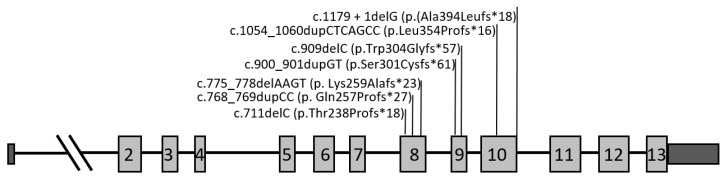
Schematic representation of the *GRN* mutations identified in this study. Untranslated regions are illustrated by dark gray boxes, and exons are shown as light gray boxes.

A total of 257 FTD patients and 19 family members were investigated for the presence of pathogenic variants in *GRN* gene. The genetic analysis revealed seven different pathogenic variants (p.Thr238Profs*18, p.Gln257Profs*27, p.Lys259Alafs*23, p.Ser301Cysfs*61, p.Trp304Glyfs*57, p.Leu354Profs*16, p.Ala394Leufs*18) in the *GRN* gene in 46 subjects including 40 patients and 6 asymptomatic family members (Figure 1). The two most common variants were p.Ser301Cysfs*61 and p.Gln257Profs*27, present in 52% (24/46) and 17% (8/46) of the subjects carrying *GRN* mutations, respectively.

**Figure 2:**
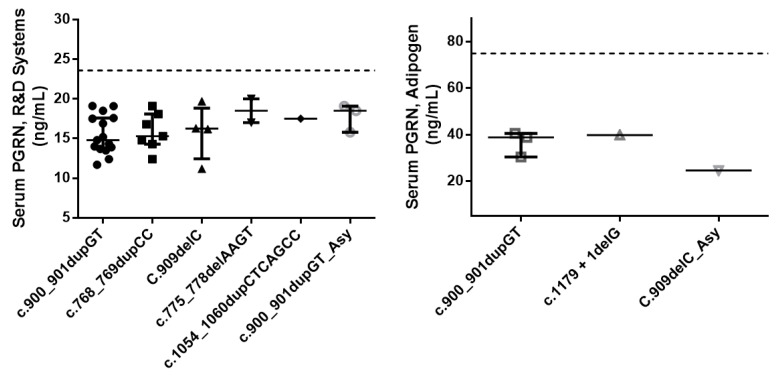
Serum PGRN levels in the studied cohort determined by the two ELISA kit used (**left**: R&D Systems; **right**: Adipogen), according to the type of mutation and clinical status. Data are presented in points as individual values and the spread of the distribution with quartiles and the median. The dotted line represents the cut-off value for each ELISA kit. Abbreviations: PGRN = progranulin; Asy = asymptomatic.

**Figure 3:**
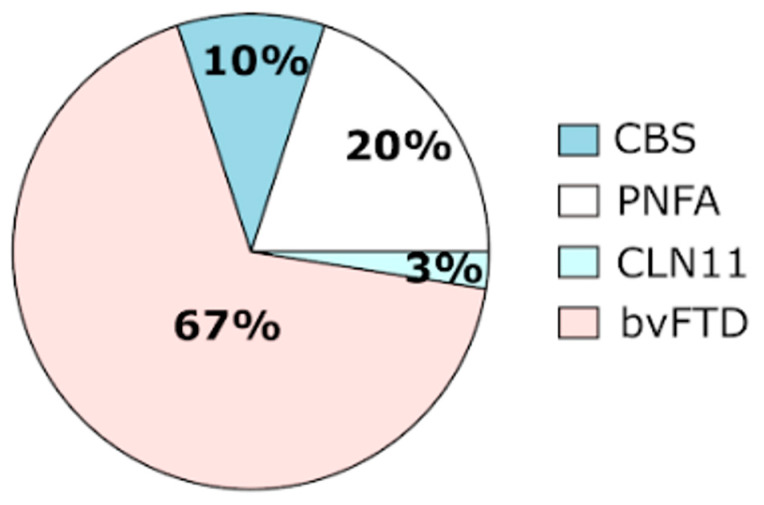
Pie chart showing the clinical presentations of the patients with *GRN* mutations.

The demographic characteristics and the respective mean serum PGRN levels are shown in Table 1. There were no differences between groups regarding gender distribution, but FTD patients were slightly older than family members (*p* = 0.003). As expected, all of the 39 subjects (35 patients and 4 family members) available for serum PGRN quantification harboring a pathogenic variant showed serum progranulin levels below the established cut-off values, supporting the pathogenic nature of these mutations (Figure 2). There were no difference in serum progranulin levels between patients and family members carrying GRN mutations (*p* = 0.235). However, due to the small number of family members tested, the comparison of serum PGRN levels between these two groups should be interpreted with caution. Of the FTD patients with GRN mutations, 8 presented with the PNFA subtype, 4 presented with CBS, and the remaining 27 presented with the bvFTD subtype (Table 2 and Figure 3). The homozygous patient had an adult-onset neuronal ceroid lipofuscinosis phenotype (CLN11). This later patient showed a PGRN serum level below the limit of detection of the R&D Systems assay (6 ng/mL) (Table 1).

Neuropsychological assessment in PNFA patients showed marked grammatical production deficits together with apraxia of speech. Mild cognitive deficits were also found in working memory, verbal initiative, planning, and dual-tasking, with relatively preserved episodic memory and visuospatial functions. Regarding bvFTD, we found a global pattern of moderate-to-severe frontotemporoparietal deficits as previously described by our group [39].

All of the seven pathogenic variants identified consisted of insertion-deletions (indel) located in exons 8 to 10, causing frameshifts that introduced premature termination codons, which result in nonsense-mediated mRNA decay (NMD) and subsequently in progranulin haploinsufficiency. Interestingly, two of these mutations were novel (c.711delC; p.Thr238Profs*18 and c.1054_1060dupCTCAGCC; p.Leu354Profs*16) and three have only been described thus far in Portuguese FTD patients (p.Ser301Cysfs*61, p.Trp304Glyfs*57, Ala394Leufs*18) (Table 2) [23,36,37,38]. The remaining two mutations identified (p.Gln257Profs*27, p.Lys259Alafs*23) were also previously reported in other populations worldwide [23,31,32,33,34,35].

Notably, all of the distinctive frameshift mutations identified were considered deleterious both by disease databases (HGMDpro, ClinVar) and annotation tools for pathogenicity predictions (VarSome, Franklin/Genoox) (Table 3). Also, all mutations except one (p.Gln257Profs*27; genomAD = 0.000004) were absent in any of the population databases (genomAD, 1000GP, ExAC) and thereby have been classified as pathogenic or likely pathogenic according to the current ACMG/AMP 2015 guidelines [40].

Furthermore, seven polymorphic variants previously described in dbSNP were found in the coding region of the *GRN* gene. Of those, two were synonymous: rs794729670 (p.Tyr294=), rs25646 (p.Asp128=) and six were non-synonymous: rs139272628 (p.His96Arg), rs25647 (p.Gly515Ala), rs748147151 (p.Pro34Thr), rs63750412 (p.Arg433Trp), rs63751100 (p.Arg418Gln), rs63750787 (p.Arg212Gln).

## 3. Discussion

Herein, a total of 276 subjects (including 257 FTD patients and 19 asymptomatic relatives) recruited over the last 10 years were investigated for the presence of *GRN* mutations. The clinical and neuropsychological phenotype of the patients carrying a pathogenic mutation was also evaluated.

Seven different pathogenic variants in the *GRN* gene were identified in 46 subjects, including 40 patients and 6 asymptomatic relatives. Of these, two were novel and five were previously described, although three of them only in the Portuguese population [23,31,32,33,34,35,36,37,38]. In our FTD cohort, the clinical diagnosis of bvFTD was three times more common than PNFA among the *GRN* mutation patients, whereas CBS was clinically diagnosed in only four cases and CLN11 in one case. No SD cases were diagnosed. A clustering of behavioral symptoms in particular, social conduct impairment/disinhibition, loss of insight, and inflexibility were the most frequent clinical features observed at disease onset. Regarding the main demographic characteristics of the patients harboring *GRN* mutations, there was approximately equal numbers of men and women, in contrast to the overrepresentation of women, commonly observed by others in *GRN* mutation groups [19]. In our study, we also found a slightly lower age at symptom onset (mean 56.0 years, SD 7.8) than that of the other groups (mean 61.3 years, SD 8.8) [19]. All of the pathogenic variants identified consisted of frameshift mutations leading to the generation of a premature stop codon that activated NMD [15,16]. Therefore, the identified mutations are believed to act through a haploinsufficiency mechanism due to the mutant mRNA degradation of the one allele, resulting in reduced levels of PGRN. Indeed, in the present work, all the subjects, either symptomatic or asymptomatic family members, harboring a pathogenic variant in the *GRN* gene showed a significant reduction in serum PGRN levels (Table 1). Of note, the asymptomatic mutation carriers showed average PGRN serum levels comparable to the patients with *GRN* mutations (Table 1), which supports the utility of the progranulin serum dosage assay for the early identification of at-risk asymptomatic subjects. Therefore, this method has been routinely used in our laboratory since 2014 and it has significantly decreased the required genetic sequencing workload. Moreover, variants’ pathogenicity was also corroborated by population databases (GenomAD, 1000GP, ExAc), disease databases (HGMD, ClinVar), and variant pathogenicity prediction tools (Varsome, Franklin/Genoox) (Table 1). Although the *GRN* mutations reported in the literature are spread throughout the coding region of the gene, in the present work all of the pathogenic variants identified appear to be clustered on exons 8, 9 and 10 (Table 2). Likewise, the most common mutation found was p.Ser301Cysfs*61, present in more than half of the subjects carrying *GRN* mutations. Thus far, this mutation has been reported only in the Portuguese population [23,36,37]. Similarly, four other mutations, p.Trp304Glyfs*57, p.Thr238Profs*18, p.Leu354Profs*16, and p.(Ala394Leufs*18), have also been detected only in Portuguese patients [23,38]. Of these, two were novel mutations, emphasizing to some extent the population-specific mutational spectrum of *GRN*. This is in line with the geographic variability observed in the distribution of *GRN* mutations in other countries and regions. Indeed, in Italy, *GRN* mutations are the most common cause of genetic FTD [41], mainly due to a large founder family with the T272fs variant [42]. Similarly, there are large *GRN* founder families in Spain (IVS7-1G > A, in the Basque country) [43,44], as well as in Belgium (IVS1 + 5G > C) [45,46].

Curiously, the p.Ser301Cysfs*61 variant was identified in 24 individuals, including 20 patients (12 bvFTD, 3 PNFA, 4 CBD, 1 CLN11) and 4 asymptomatic relatives, which emphasizes the clinical phenotype heterogeneity previously observed in patients carrying the same *GRN* mutation. This variant was present in heterozygosity in all cases except the one with the clinical presentation of CLN11. Part of the family of this latter case was firstly reported in 2008, in two affected siblings who presented with CBS in their fifth decade of life, confirming that CBS was part of the phenotype of *GRN* mutations [36]. Interestingly, years later, in 2016, our group had the opportunity to study the entire family and identified a homozygous case for this *GRN* family mutation, confirming the diagnosis of CLN11 [37]. Indeed, this patient presented at age of 25, a rapidly progressive visual deficit leading to incapacitating amaurosis within 3 years and later on, developed progressive disequilibrium and dysarthria. In contrast, heterozygous relatives presented bvFTD and some also developed extrapyramidal features compatible with CBS, highlighting the pleiotropic effect of the mutation in heterozygous or homozygous status, also reported by others [47].

The variants p.Gln257Profs*27 and p.Lys259Alafs*23 have previously been reported in several FTD patients worldwide [31,32,33,34,35]. In our cohort, p.Gln257Profs*27 was the second most common mutation, identified in 17% of the *GRN* mutation carriers. Four patients presented with bvFTD and another four with PNFA. This variant, p.Gln257Profs*27, was initially reported in 2012 in a proband who had an age at onset of 60.5 years and was diagnosed with probable AD, underscoring the occurrence of *GRN* in individuals with a clinical presentation indistinguishable from that of typical AD [31] and often with biomarkers of AD. One year after, this mutation has been reported in a Portuguese 50-year-old woman with CBS and in a family with progressive aphasia and behavioral features [32]. Later in 2017, this mutation has been seen in homozygosity in a 25-year-old female, born from consanguineous parents, who presented with a complicated spastic paraplegia. At 15 years old, the patient developed a progressive vision loss leading to blindness by the age of 19, when she also developed focal non-motor (visual) bilateral tonic–clonic seizures as well as non-motor aware seizures with visual symptoms. Her memory and executive functions declined from the age of 19. At age 20, she also developed progressive ataxia and dysarthria, leading to severe disability [33]. In 2020, this mutation was again observed in homozygosity in a female patient with CLN11 with seizures. At age 16, the proband presented generalized tonic–clonic seizures and, at age 19, she developed bilateral visual loss caused by retinitis pigmentosa with cystoid macular edema (bilateral visual acuity 2/10) and bilateral cataracts. Cerebellar gait disorder and dysarthria progressively developed at age 22. At age 25, she presented visual hallucinations and the cerebellar ataxia worsened, with saccadic pursuit, nystagmus, severe dysarthria, and dysphagia [34].

The p.Lys259Alafs*23 mutation was identified in our cohort in two unrelated female patients with the bvFTD subtype at the age of 56 and 54, respectively. One with memory, language, and behavior impairment and the other with predominant behavior changes. Both had frontotemporal atrophy, one symmetrical and the other with mild left frontotemporoparietal atrophy. This mutation had firstly been reported in 2016 in a male patient with a family history of FTD, who presented with word-finding difficulties at age 69. Slow deterioration of naming was recorded, with increasing interference in his professional activities. Apathetic behavior emerged one year after first examination, followed by disinhibition six months later. This clinical picture worsened progressively. MRI revealed marked atrophy of the anterior and medial parts of the left temporal lobe. He was diagnosed with bvFTD. Three years after first examination, he scored 11/30 on the MMSE, with marked reduction in verbal fluency [35].

In our study, we also identified two novel mutations, p.Thr238Profs*18 and p.Leu354Profs*16, in two female patients presenting with the bvFTD subtype.

The p.Thr238Profs*18 mutation was identified in a patient who had an age at onset of 59 years and presented first with cognitive impairment with predominant frontal involvement, with alterations in executive functions and behavior, with poor critical judgment and hyperorality. She also had problems with memory and disorientation, evolving towards a multi-domain cognitive impairment. Later, she developed a left-predominant rigid akinetic parkinsonism.

The second novel mutation, p.Leu354Profs*16 was identified in a patient who had an age at onset of 66 years. She started showing disinhibition, with unreasonable laughter and childish behavior. She also showed executive dysfunction, with diminished critical ability, perseveration, and apraxia. Later, she developed aggressive behavior and, later, parkinsonism.

The mutation p.Trp304Glyfs*57, which has been reported only in Portuguese patients, was present in three bvFTD cases, one PNFA, and two asymptomatic relatives. The PNFA patient developed symptoms from the age of 50, and had predominantly left frontotemporal atrophy. The bvFTD patients had symptoms from the ages of 57, 58 and 62, and all had frontotemporal symmetrical atrophy.

Interestingly, the mutation p.Ala394Leufs*18 which has been identified in our study in four bvFTD patients, has also been reported only in Portuguese patients. This mutation was initially described in 2012 in a Portuguese bvFTD patient with asymmetrical parkinsonism and prominent visuospatial deficits. This patient started developing symptoms at the age of 63 and showed right temporal atrophy [38].

Hence, it is noteworthy that in our cohort of FTD patients, *GRN* mutations have been identified in approximately 16% (40/257) of the cases and in 32% (6/19) of the asymptomatic family members. The combination of *GRN* sequencing methods with PGRN serum dosage assays has contributed greatly to the identification of subjects with *GRN* mutations in a time- and cost-effective manner. As reported by others [48], in our laboratory we also have extended the assessment of PGRN level in patients without a family history of the disease or with atypical presentations. Indeed, *GRN* mutations are also responsible for 3–5% of nonfamilial cases and for a larger phenotypic spectrum [49]. Recently, strong causal functional inferences have also been reported that connect the genetic risk of other neurodegenerative diseases to gene expression changes in the brain for PGRN [50]. This might be due to the regulatory role of progranulin on lysosomal function and inflammation, which are impaired in multiple neurodegenerative diseases; thus, maybe in the future the assessment of PGRN level should be extended to other neurodegenerative diseases rather than FTD [51,52]. Furthermore, due to the stability of peripheral PGRN levels over a long time, it is also used to monitor ongoing clinical trials based on progranulin-restoring therapy. Thus, for the 40 FTD patients in whom a *GRN* mutation has been identified, it was possible to perform 19 predictive tests in other family members, still asymptomatic, to identify the ones at high risk of developing the disease, in the context of formal genetic counselling. Indeed, *GRN* mutations have been identified in one third of the cases (32%). These results were extremely important, not only to disclose the clinical characteristics and the *GRN* mutation spectrum of the patients from the central/north region of Portugal but also to allow their enrollment (both asymptomatic-*GRN* mutation carriers and symptomatic-*GRN* mutation patients) in an ongoing clinical trial which is testing a new gene-specific therapy that aims to normalize PGRN deficiency. In fact, our center has been participating in Alector’s AL001 clinical trial where patients received AL001, a human monoclonal antibody that blocks sortilin (*SORT1*), a PGRN lysosomal trafficking receptor, preventing PGRN degradation, and thereby increasing the half-life of PGRN and elevating the level of PGRN in the brain and serum from twofold to threefold. The Phase 1 and 2 studies of AL001 are completed, and the Phase 3 study of AL001 (NCT04374136) to evaluate the safety and effects of the experimental drug AL001 in people with or at risk for FTD due to a mutation in the GRN gene is ongoing.

In conclusion, the frequency of *GRN* mutations in our patients’ cohort was 16%, whereas bvFTD was the most common clinical presentation among the *GRN* mutation carriers (69%), and among those who underwent neuropsychological evaluation, we found a neurocognitive profile compatible with a global pattern of moderate-to-severe frontotemporoparietal deficits. Two novel null *GRN* mutations were identified (c.711delC; p.Thr238Profs*18 and c.1054_1060dupCTCAGCC; p.Leu354Profs*16), with strong evidence of pathogenicity. This study broadens the mutation spectrum of GRN and provides an update of the molecular basis of the FTD cohort from the central/north region of Portugal. The identification of the underlying GRN mutations has been essential to provide accurate genetic counselling and clinical management. Finally, it also allowed the enrolment of subjects with *GRN* mutations (both asymptomatic and symptomatic) in ongoing clinical trials, which may offer new hope for patients and their families.

## 4. Material and Methods

### 4.1. Participants

During the period between January 2013 and January 2023, a total of 276 subjects were investigated for the presence of pathogenic variants in the *GRN* gene at the Neurogenetics Laboratory of the Center for Neuroscience and Cell Biology (CNC) at the University of Coimbra. Of these, 257 subjects had a clinical presentation within the spectrum of FTD and 19 were asymptomatic family members who provided their written informed consent for being tested. Patients were referred from the Departments of Neurology of Coimbra Hospital and University Centre (CHUC), University Hospital Center of São João (UHCSJ), and the Local Health Unit of Matosinhos. All of the unaffected family members were referred from the Genetic Counseling Unit at the Medical Genetics Department of CHUC. All of the patients were in a stable condition, without acute comorbidities, and underwent a thorough biochemical and neurological evaluation performed by a behavioral neurologist. For all of the patients, a detailed history, clinical neurological examination, psychiatric evaluation, neuropsychological assessment, brain imaging (CT or MRI and SPECT), and genetic testing were performed.

### 4.2. Clinical and Neuropsychological Evaluations

The diagnosis of FTD was based on the Lund and Manchester clinical criteria [53,54], revised by the Work Group on Frontotemporal Dementia and Pick’s Disease [55], and more recently according to the International Behavioural Variant Frontotemporal Dementia Criteria Consortium for bvFTD [56] and the proposed criteria for primary progressive aphasia [57]. The comprehensive neuropsychological assessment included the Mini Mental State Examination (MMSE) [58], the Montreal Cognitive Assessment (MoCA) [59]; and the Battery of Lisbon for the Assessment of Dementia (BLAD) [60]. This battery includes some tests of the Wechsler Memory Scale (WMS) [61] and comprises the following cognitive abilities: attention (Cancellation Task); verbal initiative (Semantic Fluency), motor and graphomotor initiatives; verbal comprehension (a modified version of the Token Test); verbal and nonverbal reasoning (Interpretation of Proverbs and the Raven’s Coloured Progressive Matrices—Ab series); orientation (spatial, temporal, and social orientation); visuoconstructive abilities (cube copy); basic written calculus; immediate memory (Digit Span Forward); visual memory (WMS Visual Reproduction Test); working memory (Digit Span Backward); learning and verbal memory (WMS Verbal Paired-Associate Learning, Logical Memory and Word Recall). All tests were administered and scored according to standardized procedures. Individual test scores were converted into z-scores. The presence of impairment is considered when the z-score < −1.

### 4.3. Laboratory Determinations

#### 4.3.1. GRN Sequencing Analysis

Blood samples from all individuals were collected into EDTA tubes with subsequent isolation of the DNA using the NZY tissue gDNA Isolation kit (Nzytech, Lisbon, Portugal), as described by the manufacturer. The genetic analysis of the *GRN* gene (NM_002087.3) was initially performed by Sanger sequencing in which all *GRN* exons and a minimum of 20 base pairs of intronic region flanking each exon were amplified by PCR, as previously described by our group [23]. Then, the PCR products were purified using the ExoSAP-IT method (Isogen life science, Utrecht, The Netherlands) and subsequently sequenced on a capillary automated sequencer CEQ 8000 (Beckman Coulter, Brea, CA, USA). The sequence analysis was performed using Sequencher 5.2 software. More recently, the *GRN* gene was sequenced on the Illumina MiSeq sequencer using an NGS-customized gene panel developed by our group. Library preparation was performed using the Agilent QXT Target Enrichment system, according to the manufacturer’s protocols. Bioinformatic analysis was used to perform the reading, alignment, and variant calling through the SureCall 4.2 software (Agilent, Santa Clara, CA, USA). Identified variants were evaluated for coverage and visually inspected using the Integrative Genomics Viewer. Variant annotation was performed using a multistep process workflow to individually assess variants’ pathogenicity, based on the guidelines for variant interpretation of the American College of Medical Genetics and Genomics and the Association for Molecular Pathology (ACMG/AMP) 2015 guidelines [40]. Population databases used for analysis included the 1000 Genomes project (1000GP), Exome Aggregation Consortium (ExAC), and Genome Aggregation Database (gnomAD). Disease databases (Human Gene Mutation Database-HGMD Professional, http://www.hgmd.cf.ac.uk/ac/ accessed on 2 October 2023 and ClinVar, https://www.ncbi.nlm.nih.gov/clinvar accessed on 2 October 2023), a variant database (dbSNP), and annotation tools for investigating variant pathogenicity (VarSome, https://varsome.com/ accessed on 2 October 2023 and Franklin/Genoox, http://franklin.genoox.com accessed on 2 October 2023) were also used.

#### 4.3.2. PGRN Level Determination

Blood samples from 39 subjects harboring a pathogenic variant (35 patients and 4 family members) were available for serum PGRN level assessment to confirm the pathogenicity of the identified variants. The blood samples were collected into serum separation tubes for PGRN quantification. These tubes were left at room temperature for 30 min to clot and centrifuged at 1500× *g* at 4 °C for 10 min. Serum was separated and frozen at −80 °C until analysis. The PGRN level was assessed by commercial ELISA kits, using either the R&D Systems (Minneapolis, MN, USA) or the Adipogen (Seoul, Korea) kits, as previously described [24]. All samples were assessed in duplicate and a positive (*GRN* mutation carrier) and negative (no *GRN* mutation) control sample were included in each run for internal quality control. According to previously determined cut-off levels for each kit [24], PGRN serum levels in each sample were classified as normal/abnormal.

As expected, all the 39 subjects (35 patients and 4 family members) available for serum PGRN quantification, harboring a pathogenic variant showed serum progranulin levels below the established cut-off values, supporting the pathogenic nature of these mutations.

### 4.4. Statistical Analysis

Statistical analyses were performed using the Statistical Package for the Social Sciences, version 20.0 (IBM SPSS, Inc., Chicago, IL, USA). To test for normal distribution, the Shapiro–Wilk test was used. As age and serum PGRN levels were not normally distributed, differences between groups were analyzed using Mann–Whitney U test. Fisher’s exact test was used for the comparison of gender distribution between groups. All tests were two-tailed and a *p*-value < 0.05 was assumed to be statistically significant.

## Figures and Tables

**Table 1:** Demographic characteristics of the subjects with *GRN* mutation and the respective serum PGRN levels.

	FTD Patients	Family Members	*p*-Value
*n*	39	6	
Age, years	57.0 [53.0–62.0]	38.5 [35.3–45.3]	0.003
Gender F/M	20/19	3/3	0.625
Serum PGRN, ng/mL			
R&D Systems (*n* = 30/3/1)	15.9 [14.0–17.7]	18.5 [17.2–18.8]	0.235
Adipogen (*n* = 4/1)	39.3 [34.6–40.1]	24.6	

Data for age and serum progranulin are represented as the median [interquartile range]. Cut-off values for serum PGRN values are 23.6 ng/mL for the R&D Systems kit and 74.9 ng/mL for Adipogen kits.

**Table 2:** *GRN* (NM_002087.3) mutations identified in our study cohort.

Nucleotide Change	Predicted Protein Change	Location (Exon/Intron)	Total Number of Cases	Zygosity	Clinical Presentations	References
c.711delC	p.Thr238Profs*18	Exon 8	1	Heterozygous	bvFTD	Novel
c.768_769dupCC	p.Gln257Profs*27	Exon 8	8	Heterozygous	bvFTD (*n* = 4)	[23,31,32,33,34]
					PNFA (*n* = 4)	
c.775_778delAAGT	p.Lys259Alafs*23	Exon 8	2	Heterozygous	bvFTD (*n* = 2)	[35]
c.900_901dupGT	p.Ser301Cysfs*61	Exon 9	23	Heterozygous	bvFTD (*n* = 12)	[23,36,37]
					PNFA (*n* = 3)	
					CBS (*n* = 4)	
					Asymptomatic (*n* = 4)	
			1	Heterozygous	CLN11 (*n* = 1)	[37]
c.909delC	p.Trp304Glyfs*57	Exon 9	6	Heterozygous	bvFTD (*n* = 3)	[23]
					PNFA (*n* = 1)	
					Asymptomatic (*n* = 2)	
c.1054_1060dupCTCAGCC	p.Leu354Profs*16	Exon 10	1	Heterozygous	bvFTD	Novel
c.1179 + 1delG	p.(Ala394Leufs*18)	IVS10	4	Heterozygous	bvFTD (*n* = 4)	[23,38]

**Table 3:** *GRN* variants analysis using different databases and annotation tools for pathogenicity prediction.

***GRN* Gene**	**Variant**	**Variant Database**	**Disease Databases**		**Tools to Predict Pathogenicity**	
		**dbSNP**	**HGMD**	**ClinVar**	**VarSome**	**Franklin/Genoox**
	p.Thr238Profs*18	-	-	Pathogenic	Pathogenic	Pathogenic
	p.Gln257Profs*27	rs1567887004	CI127616 DM	Pathogenic	Pathogenic	Pathogenic
	p.Lys259Alafs*23	-	CD164461 DM	Pathogenic	Pathogenic	Pathogenic
	p.Ser301Cysfs*61	-	CI085841 DM	-	Pathogenic	Likely pathogenic
	p.Trp304Glyfs*57	rs63750366	CD077404 DM	-	Pathogenic	Likely pathogenic
	p.Leu354Profs*16	-	-	-	Likely pathogenic	Likely pathogenic
	p.(Ala394Leufs*18)	-	CD124917 DM	-	Likely pathogenic	Likely pathogenic

Key: - no data available.

## Data Availability

Data are contained within the article.
